# In time: the persistence of congenital syphilis in Brazil - More progress needed!

**DOI:** 10.1016/j.rppede.2016.06.004

**Published:** 2016

**Authors:** Joshua M. Cooper, Ian C. Michelow, Phillip S. Wozniak, Pablo J. Sánchez

**Affiliations:** a Nationwide Children's Hospital, The Ohio State University College of Medicine, Columbus, EUA; b Rhode Island Hospital, Alpert Medical School of Brown University, Providence, United States

Despite decades of epidemiologic and clinical experience with maternal and congenital syphilis, both remain major public health problems in Brazil and in the rest of the Americas. In 2010 and supported by the World Health Organization (WHO), the Pan American Health Organization (PAHO) Member States approved the Strategy and Plan of Action for the Elimination of Mother-to-Child Transmission of HIV and Congenital Syphilis with the goal of reducing the incidence of congenital syphilis to ≤0.5 cases per 1000 live births by 2015.^1^ In 2014, 17,400 cases (1.3/1000 live births) of congenital syphilis were reported in the Americas, and 17 countries may have eliminated maternal-to-child transmission of syphilis.^2^ Despite some progress, Brazil did not meet the congenital syphilis elimination goal but rather, the epidemic rages on, resulting in substantial fetal and neonatal mortality. In 2010, 6916 cases (2.27/1000 live births) of congenital syphilis were reported to the Brazilian Ministry of Health and PAHO, while in 2013, the number of cases increased to 13,705 (4.70/1000 live births) before decreasing to 6793 cases in 2014.^2^^,^ 3

Congenital syphilis is a preventable disease, and there must be zero tolerance for its occurrence as even one case represents a failure of the public health system. Health care professionals know what must be done to prevent congenital syphilis and its complications which include stillbirth, prematurity, nonimmune hydrops fetalis, and neonatal mortality.^4^ The WHO estimates that globally, 1.5-1.85 million pregnant women are infected with syphilis annually and half of them have infants with adverse outcomes.^4^ In the United States from 1999 to 2013, neonatal mortality secondary to congenital syphilis was 12/1000 live births, with a case fatality rate of 6.5%.^5^ Of the 418 reported deaths, 82% were stillbirths and 89% of the mothers had untreated or inadequately treated syphilis. Moreover, less prenatal care was associated with increased risk of death, and importantly, 59% of the deaths occurred by 31 weeks of gestation.

It is clear that pregnant women must have access to early prenatal care and be screened serologically for syphilis at the first prenatal visit and, in high risk areas, again at 28-32 weeks’ gestation and delivery.^6^ According to PAHO, 94% of pregnant women in the Americas attended at least one antenatal care visit during the pregnancy, and 80% received syphilis testing at some point during the pregnancy.^2^ In Brazil, Domingues et al.^7^ interviewed 23,894 women postpartum and reported that 98.7% had at least one antenatal care visit, 89% had documentation of at least one syphilis test on prenatal record cards, but only an additional 41% had a second test performed. From 2011 to 2014, PAHO reported an increase from 81% to 86% in syphilis-infected women who had documentation of appropriate treatment, although it was still below its goal of 95%.^2^ It therefore is not surprising that congenital syphilis remains a major problem in Brazil and the rest of the Americas.

In addition to identification of infected pregnant women, timely treatment is mandatory for prevention of congenital syphilis.^8^^,^ 9 In locales where follow-up is uncertain or difficult, rapid point-of-care syphilis testing should be performed so that women are treated on site and without delay. In addition, serologic testing and presumptive treatment of their sexual partner is essential to prevent reinfection and transmission to the fetus.^10^ In Brazil, it has been estimated that only about 12% of sexual partners received treatment for syphilis,^11^ certainly a failure of the public health infrastructure as contact tracing and treatment is the major method of controlling syphilis transmission in communities.

Penicillin G is the only known effective antimicrobial agent for preventing vertical transmission of syphilis and treating fetal infection.^6^ Pregnant women should receive the penicillin regimen appropriate for the stage of infection, and if any dose of therapy is missed for latent syphilis, the full course of therapy must be repeated. Pregnant women who have a history of penicillin allergy should be desensitized and treated with penicillin.

Unfortunately, the diagnosis of congenital syphilis remains problematic due to the inability to detect or culture *Treponema pallidum* in clinical specimens, thus necessitating reliance on laboratory tests that detect maternal nontreponemal and treponemal IgG antibodies transferred transplacentally to the fetus. Nonetheless, the use of IgM immunoblotting, PCR assays, and rabbit infectivity testing (RIT, inoculation of infected patient fluid into rabbit testes with resultant syphilitic infection of the rabbit) in research laboratories has allowed evidence-based rationale for the management of infants born to mothers with reactive serologic tests for syphilis.^12^^-^ 15

Neonates with proven or highly probably syphilis, that is, those who have an abnormal physical examination, serum quantitative nontreponemal serologic titer that is fourfold or higher than the mother's titer, or positive darkfield microscopy or PCR of lesions or body fluids/tissues/placenta,^16^ are diagnosed readily and should receive 10 days of intravenous aqueous crystalline penicillin G or intramuscular procaine penicillin G therapy. Virtually all of these infants have a positive IgM immunoblot, and at least 50% of them have spirochetes detected in cerebrospinal fluid by RIT.^17^

The well-appearing infant with a normal physical examination and born to a mother with untreated or inadequately treated (<4 weeks before delivery or any nonpenicillin G regimen) syphilis remains a diagnostic conundrum. However, while as many as 20% of these infants have a positive IgM immunoblot indicative of *in utero* infection, almost none will have central nervous system invasion by *T. pallidum* if their complete evaluation (complete blood cell count and platelets, long bone radiographs, and cerebrospinal fluid [CSF] examination) is normal.^17^ These infants can receive a single intramuscular injection of benzathine penicillin G (50,000U/kg).^6^ Finally, normal infants born to mothers adequately treated during pregnancy and greater than 4 weeks before delivery should be considered as a “close contact” and receive a single intramuscular injection of benzathine penicillin G, although no evaluation is required or recommended.^6^ Similarly, normal infants who have a nonreactive serum nontreponemal test result but are born to mothers with untreated or inadequately syphilis can receive a single dose of intramuscular benzathine penicillin G without evaluation - an increasingly common scenario with the use of treponemal tests such as enzyme immunoassays or chemiluminescence immunoassays for syphilis screening (“reverse sequence” screening).^18^

As syphilis can be a co-factor for HIV infection, all women and their sexual partner(s) who have syphilis should be tested for HIV infection. Infants born to mothers coinfected with syphilis and HIV do not require different evaluation, therapy or follow-up.

All infants with reactive nontreponemal tests should receive careful follow-up examinations and serologic testing (i.e., a nontreponemal test) every 2-3 months until the test becomes nonreactive. A reactive serum treponemal test beyond 18 months of age when maternal antibodies have disappeared confirms a diagnosis of congenital syphilis, although as many as 20% of infected infants may serorevert completely to nonreactive syphilis serologic tests.

Recently, a penicillin shortage in Brazil and other parts of the world has posed a serious health threat to fetuses and infants of mothers with syphilis. If preparations of penicillin are unavailable, a 10 day course of ceftriaxone can be considered with careful clinical and serologic follow-up, including repeat CSF evaluation.^6^^,^ 19 Research efforts are needed to evaluate whether other antibiotics such as ampicillin can treat effectively central nervous system disease.

The lack of timely identification and appropriate treatment of infected infants can have profound consequences in later life. Manifestations of late congenital syphilis involve the central nervous system, bones and joints, teeth, eyes, and skin and include Hutchinson's triad (interstitial keratitis, eighth cranial nerve deafness, notched central incisors), named after Sir Jonathan Hutchinson (1828-1913) from England.

Research and humanitarian efforts must continue to control, treat, and eventually eliminate congenital syphilis globally. The public health impact of syphilis in pregnancy and infancy remains substantial, and only through optimal prenatal healthcare services will elimination of maternal-to-child transmission of syphilis become a reality in the Americas.
